# Evidence for a Clinically Meaningful Effect of no-Touch Harvesting Technique on Vein Graft Patency—A Bayesian Reanalysis of the SWEDEGRAFT Trial

**DOI:** 10.1093/ejcts/ezaf407

**Published:** 2025-11-13

**Authors:** Ulrik Sartipy, Samuel Heuts, Stefan K James

**Affiliations:** Department of Cardiothoracic Surgery, Karolinska University Hospital, Stockholm, Sweden; Department of Molecular Medicine and Surgery, Karolinska Institutet, Stockholm, Sweden; Department of Cardiothoracic Surgery, Maastricht University Medical Centre, Maastricht, The Netherlands; Cardiovascular Research Institute Maastricht (CARIM), Maastricht University, Maastricht, The Netherlands; Uppsala Clinical Research Center, Uppsala University, Uppsala, Sweden; Department of Medical Sciences, Uppsala University Hospital, Sweden, Cardiology, Uppsala

We reanalyzed the SWEDEGRAFT randomized trial using a Bayesian framework that combined literature-informed priors with the observed trial data. Posterior inference suggests that no-touch (NT) saphenous vein harvesting likely reduces the risk of vein graft failure compared with conventional harvesting, although the probability of achieving a clinically meaningful absolute risk reduction (ARR) depends strongly on baseline risk and the prespecified minimal clinically important difference (MCID). The probability of any benefit was high (∼97%), whereas the probability of an ARR ≥4% was moderate (∼62%) and fell when the MCID was raised to 5% (∼45%). At a 15% baseline risk, the probability of achieving an ARR ≥5% is very small. This reanalysis demonstrates moderate to low evidence for a clinically relevant effect of NT vein harvesting on the risk of vein graft failure in the SWEDEGRAFT trial.

SWEDEGRAFT was a registry-based, binational, randomized controlled trial in which 902 patients were allocated to NT or conventional saphenous-vein harvesting.^1^^,^^2^ Over a mean follow-up of 3.5 years, the primary composite outcome (death, occlusion or >50% vein graft stenosis, or vein graft intervention) occurred in 19.8% of NT patients vs 24.0% with conventional harvesting (absolute difference, −4.3%; 95% CI −10.1 to 1.6; *P* = .15). Using a frequentist analysis, the trial concluded that NT was not superior to conventional open harvesting in reducing vein graft failure.

Thus, SWEDEGRAFT observed a 4.3% absolute risk difference favoring NT, though not meeting the conventional thresholds for statistical significance—an apparently paradoxical but common scenario in “neutral” trials, which illustrates the difficulty of interpreting results when power is limited. The trial was criticized as underpowered, partly because it assumed a 50% 2-year failure rate in the conventional arm, and some argued that the neutral result likely reflects a type II error in the presence of a true benefit of NT.^3^ In simpler terms: “with a larger sample, the between-group difference might have reached statistical significance”, thus turning a “negative/neutral” into a “positive” trial, despite a similar absolute difference.

Are neutral, underpowered trials useless? No, but their interpretation requires afterthought and a broad understanding of the clinical context. Bayesian methods offer a complementary view by quantifying the probability that an effect in a certain direction exists and that it exceeds a clinically relevant threshold—information that is more actionable for decision-making than simply reporting that “the null hypothesis could [or could not] be rejected”. Briefly, Bayesian analysis combines prior knowledge of the expected/known treatment effect with the observed trial data to compute the posterior probability distribution of the effect.^4^^,^^5^ From this distribution, one can answer questions such as “What is the probability the intervention works?” and, more importantly, “What is the probability that the intervention produces a clinically meaningful effect?”

We therefore performed a Bayesian reanalysis of SWEDEGRAFT to provide a clinically oriented interpretation of its primary outcome.

A detailed description of the analyses and results are available in the Supplementary online material (https://ulriksartipy.github.io/SWEDEGRAFT_Bayesian_Reanalysis)

We constructed a hierarchical, literature-based prior by pooling evidence from one randomized controlled trial^6^ and one network meta-analysis^7^ under weakly informative assumptions. The pooled estimate (mean OR 0.65; 95% credible interval 0.33-1.45) served as the prior for reanalyzing the SWEDEGRAFT data.

The posterior probability of *any* beneficial effect of NT on vein graft failure was 96.7%, indicating a very high probability that NT improves graft outcomes.

To assess clinical relevance, we specified a 4% ARR as the MCID—a conservative choice given the burden of leg-wound complications, and that the primary end-point was a surrogate for hard clinical outcomes. For context, the PATENCY trial^6^ was powered to detect a 43% relative risk reduction from an 8.4% control risk, which translates to a 4.1% ARR at 1 year. Using the SWEDEGRAFT control-arm risk (24% at ∼3 years), an ARR of 4% corresponds to an OR threshold of 0.79. By this criterion, the posterior probability of a *clinically relevant* effect was 62%. Sensitivity analyses using skeptical and enthusiastic priors yielded probabilities between 21% and 52% for achieving the MCID, demonstrating only moderate sensitivity to prior specification—and a consistently low-to-moderate probability of a clinically meaningful treatment effect.

Because baseline risk varies across populations—and because different health-care settings may adopt different MCIDs—we also examined ARR across a range of baseline risks. Cumulative posterior probabilities show that lower baseline risks substantially reduce the probability of exceeding a given ARR threshold; for example, at a 15% baseline risk, the probability of achieving an ARR ≥5% is consistently low. Because all data in this complementary investigation are derived from published results of the SWEDEGRAFT trial, all limitations of the original trial also apply to this reanalysis.

In conclusion, this Bayesian reanalysis of SWEDEGRAFT indicates that NT vein harvesting likely reduces graft failure, but the probability of exceeding a conservative 4% ARR is only moderate and declines further at lower baseline risks or higher MCID thresholds. In populations with baseline risk ≤15%, the probability of meeting an MCID of 5% is very low. This approach reframes a “neutral” frequentist result into clinically interpretable probability statements, supporting more nuanced decision-making when power is limited.

## Supplementary Material

ezaf407_Supplementary_Data

## Data Availability

The data underlying this article are available in the public domain. The code is available at https://ulriksartipy.github.io/SWEDEGRAFT_Bayesian_Reanalysis.
