# Supplementary material for: Evidence for a Clinically Meaningful Effect of no-Touch Harvesting Technique on Vein Graft Patency—A Bayesian Reanalysis of the SWEDEGRAFT Trial
Source: Eur J Cardiothorac Surg. 2025 Nov 13;67(12):ezaf407. doi: 10.1093/ejcts/ezaf407 (PMC12673058; doi:10.1093/ejcts/ezaf407)
Supplement: ezaf407_Supplementary_Data [file ezaf407_supplementary_data.pdf]

## Response to Reviewers

Manuscript ID: **EJCTS-2025-102002**

Manuscript title: *"Evidence for a clinically meaningful effect of no-touch harvesting technique on vein graft patency – A Bayesian reanalysis of the SWEDEGRAFT trial"*.

### Comments from the Associate Editor and Reviewers:

**Associate Editor: AE:**

**Thank you for providing me with the reviewing of the invited commentary Evidence for a clinically meaningful effect of no-touch harvesting technique on vein graft patency – A Bayesian reanalysis of the SWEDEGRAFT trial.**

**Please observe the statistical reviewer's comments.**

**Were the calculations and results based on AI or only the text and language?**

***Authors' response***
Thank you for the opportunity to revise our paper.
In this manuscript, AI was only used for text/language checking (i.e., spelling and grammar). This was specified on line 112 in the manuscript.

**Reviewer 1: STATISTICAL REVIEW**

**This Bayesian reanalysis provides a transparent, reproducible, and clinically relevant re-interpretation of the SWEDEGRAFT trial. The authors should be commended for making their data, priors, and full code openly available, and for moving beyond dichotomous p-values toward probability-based decision metrics (e.g., Pr[ARR ≥ MCID]).**

**IMHO, the manuscript would benefit from clearer definition of estimands, justification of prior transportability, and more complete reporting of model diagnostics and sensitivity analyses. Comments apply to both the manuscript and the supplemental material available at https://ulriksartipy.github.io/SWEDEGRAFT_Bayesian_Reanalysis/SWEDEGRAFT_Bayesian_Reanalysis.html**

**Major Comments**

**1. The manuscript should explicitly define the estimand for the Bayesian model (e.g., per-patient composite graft failure at 3 years). The prior distribution appears to be derived from studies using slightly different endpoints (per-graft angiographic occlusion at various follow-ups).**

**I suggest including a table mapping each prior source to endpoint definition, time horizon, and analysis scale, and discuss how differences may influence prior transportability.**

***Authors' response***

Thank you for the careful and constructive review of our paper. We are very grateful for the positive and enthusiastic feedback on our work.

As suggested by the Reviewer, we have added a Table to the supplemental online material, please see below. We agree that the heterogeneity between studies regarding e.g., endpoint definitions and follow-up duration, particularly in the network meta-analysis, is a limitation, and negatively affects prior transportability. However, we performed sensitivity analyses to investigate the effect of different priors and found that our results were fairly robust.

| Prior source | Study design | Endpoint definition | Time horizon | Analysis scale |
|---|---|---|---|---|
| Gaudino, 2021 | Network meta-analysis of randomized controlled trials. Conventional: 11 studies, NT: 5 studies | Varied between studies. The primary endpoint was graft occlusion at protocol-defined angiographic follow-up | Varied between studies. Approximatley 4 to 4.5 years | log(odds ratio)* |
| Tian, 2025 | Randomized controlled trial | Vein graft failure per patient | 3 years | log(odds ratio) |

*Crude patency rates were extracted from Table 2 in the article, and were used to calculate number of events.

**2. The web supplement presents the prior distribution graphically, but there is no formal prior-posterior overlap or prior-predictive check to evaluate potential conflict between external evidence and the SWEDEGRAFT likelihood.**

**I suggest adding plots comparing prior, likelihood, and posterior; include prior-predictive simulations to demonstrate that the observed data are plausible under the prior.**

***Authors' response***

As suggested by the Reviewer, we have added prior predictive checks to the supplemental online material, please see below. The graph demonstrate that the observed data were compatible with the chosen prior. We have presented plots comparing prior, likelihood, and posterior (Figure 2 in the supplemental online material).

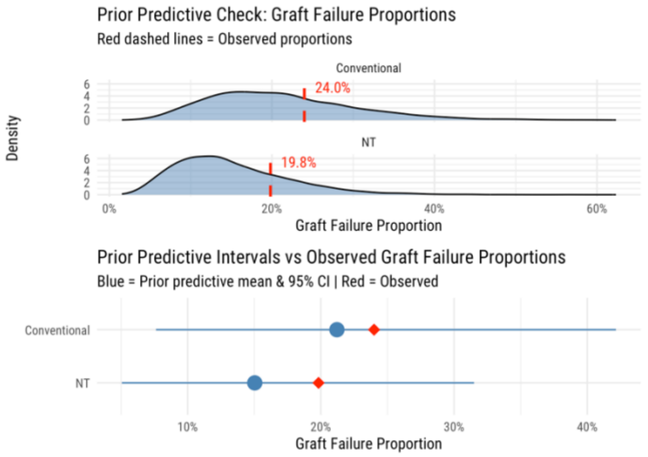

**3. The current reanalysis contrasts "enthusiastic" and "skeptical" priors but does not quantify their effective information content.**

**A prior-effective-sample-size or power-prior sensitivity analysis (varying prior weight w □ [0, 1]) to demonstrate how posterior conclusions depend on prior strength would be helpful.**

***Authors' response***

As suggested by the Reviewer, we have added a prior effective sample size analysis to the supplemental online material, please see below.

**Table 1.1 Prior Effective Sample Size Analysis**

How much information does each prior contribute to the posterior distribution, expressed as an equivalent hypothetical sample size.

| PRIOR TYPE | SD (LOG OR) | $J_{PRIOR}$ | $J_{PRIOR}/J_{DATA}$ (INFORMATION FRACTION) | % INFORMATION RELATIVE TO SWEDEGRAFT | HYPOTHETICAL TRIAL SIZE (N) |
|---|---|---|---|---|---|
| Meta-analytical | 0.3696805 | 7.32 | 0.19 | 19 | 172 |
| Skeptical | 0.1823444 | 30.08 | 0.79 | 79 | 708 |
| Enthusiastic | 0.4456207 | 5.04 | 0.13 | 13 | 119 |

Information (denoted as J) is defined as 1/variance; $J=1/SD^2$

**4. Stan/brms settings are described, but convergence and fit statistics (R-hat, ESS, divergences, posterior predictive checks) are not reported.**

***Authors' response***

All R-hat statistics were <1.05. More technical details on convergence and model fit were not reported because we would expect that most readers would not be interested. Expert readers with special interest could run the code and inspect convergence and the relevant fit statistics.

**5. Multiplicity and decision thresholds**

**The paper examines several MCID values (4% and 5%) and baseline risks. Although Bayesian inference does not require multiplicity adjustment, EJCTS readers expect a pre-specified primary decision rule. I recommend defining one primary MCID (e.g., 4%) anchored to the SWEDEGRAFT control rate and describe others as sensitivity analyses.**

***Authors' response***

We agree with the Reviewer, however, we did specify that the MCID of 4% were the primary focus on line 82 in the manuscript.

**6. The Bayesian analysis relies on published summary data rather than individual-level data; therefore, missing angiographic follow-up cannot be re-modeled.**

**I suggest clarifying that the reanalysis inherits the SWEDEGRAFT trial's multiple-imputation assumptions and suggest, if feasible, a simple sensitivity (e.g., tipping-point prior on missing outcomes) to show robustness.**

***Authors' response***
We agree that this reanalysis inherit all the limitations of the SWEDEGRAFT trial. To clarify, we have added the following text to the revised version of the manuscript:

***"Because all data in this complementary investigation are derived from published results of the SWEDEGRAFT trial, all limitations of the original trial also apply to this reanalysis."***

**Minor Comments**

**1. The text appropriately emphasizes clinical interpretation, but avoid suggesting causality beyond the scope of reanalysis.**

***Authors' response***
The purpose of this reanalysis of the SWEDEGRAFT trial was to provide a clinically oriented interpretation and in essence, answer the question "What is the probability that the NT technique has a clinically meaningful effect?". We believe that we have accomplished this, and our conclusions are supported by the data and analyses presented in the supplemental online material. We hope that this alternative perspective on the SWEDEGRAFT trial results will provide a foundation for further discussion within the cardiac surgery community regarding the clinical value of NT SVG harvesting in CABG.
